# Two-dimensional code enables visibly mapping herbal medicine chemome: an application in *Ganoderma lucidum*

**DOI:** 10.1186/s13020-022-00702-8

**Published:** 2023-01-12

**Authors:** Qian Wang, Wenjing Liu, Bo Peng, Xingcheng Gong, Jingjing Shi, Ke Zhang, Bo Li, Pengfei Tu, Jun Li, Jun Jiang, Yunfang Zhao, Yuelin Song

**Affiliations:** 1 Modern Research Center for Traditional Chinese Medicine, School of Chinese Materia Medica, Beijing University of Chinese Medicine, Beijing, China; 2 Amway (China) Botanical Research Center, Wuxi, China; 3 Amway (Shanghai) Innovation & Science Co., Ltd., Shanghai, China; 4 Shandong Institute for Food and Drug Control, Ji’nan, China

**Keywords:** *Ganoderma lucidum*, Two-dimensional code, “Coding-decoding” workflow, Chemical profile, Herbal medicine recognition

## Abstract

**Background:**

Chemical profile provides the pronounced evidence for herbal medicine (HM) authentication; however, the chemome is extremely sophisticated. Fortunately, two-dimensional (2D) code, as a quick response means, is conceptually able to store abundant information, exactly fulfilling the chemical information storage demands of HMs.

**Methods:**

We here attempted to denote both MS$^1$ and MS$^2$ dataset of HM with a single 2D-code chart. Measurement of *Ganoderma lucidum* that is one of the most famous HMs with LC–MS/MS was employed to illustrate the “coding-decoding” workflow for the conversion amongst MS/MS dataset, 2D-code, and chemical profile, and to evaluate the applicability as well. After data acquisition, and *m/z* value of each deprotonated molecular signal was divided into integer and decimal portions, corresponding to *x* and *y* coordinates of 2D-plot, respectively. On the other side, *m/z* values of all its fragment ions were exactly assigned to serial *x* values sharing an identical *y* value being equal to the precursor ion. 2D-code was thereafter produced by plotting these defined dots at a 2D-chart. Regarding a given 2D-code map, the entire chart (*x* coordinate: 0–600; *y* coordinate: 0–600) was fragmented into two regions by the line of *y*=*x*. MS$^1$ spectral signals always located below the line, whereas all fragment ions lay at the left zone. After extracting information from the edges of each square frame, *m/z* values of both precursor ion and fragment ions could be harvested and putatively deciphered to a compound through applying some empirical mass fragmentation rules.

**Results:**

The entire code of *Ganoderma lucidum* fruit bodies therefore corresponded exactly to a compound set. The elution program, even the employment of direct infusion, couldn’t significantly impact the code, and dramatical differences occurred between different species and amongst different parts of *Ganoderma lucidum* as well. Not only ganoderic acid cluster but also certain primary metabolites served as the diagnostic compounds towards species differentiation.

**Conclusion:**

2D-code might be a meaningful, practical visual way for rapid HM recognition because it is convenient to achieve the conversion amongst MS/MS dataset, 2D-barcode plot, and the chemome.

**Supplementary Information:**

The online version contains supplementary material available at 10.1186/s13020-022-00702-8.

## Background

It is extremely important to authenticate the original sources of herbal medicines (HMs) [1], because some HMs show quite similar appearances, and nonetheless, own completely different pharmacological properties. The phytologists traditionally authenticate HMs on the basis of their macroscopic together with microscopic features through direct or microscope-assisted observation. Obviously, the task requires plentiful prior knowledge and experiences; however, such experiences-dependent authentication is significantly challenged by much misrecognition even being aided by artificial intelligence [2]. Notably, safety accidents may happen for those confusing HMs with toxic and/or hazardous risks, *e*.*g*., Japanese star anise (*Illicium anisatum*) *vs*. Chinese star anise (*Illicium verum*) [3], and Momordicae Semen (Chinese name: *Mubiezi, Momordica cochinchinensis*) *vs*. Strycczi Semen (Chinese name:*Maqianzi, Strycczos nux-vomica*) [4, 5]. Because each species intrinsically possesses unique genome, the whole genome sequencing should be the golden authentication standard; however, it is challenging and dramatically expensive to acquire all genetic information. As a compromise, it might be viable to focus on certain diagnostic genes, for instance ITS, ITS2, and rbcL segments [6–8], and in response to this principle, DNA barcoding is now being extensively favored and applied as the fit-for-purpose approach for HM authentication [9]. However, the application of the versatile tool is critically narrowed by some shortcomings, *e*.*g*., expensive instrumentation and the option of reliable sequences, and furthermore, it is prominently challenging to extract the desired genes from the consumable HMs products, *e*.*g*., traditional Chinese medicine prescriptions.

As the genome endpoints, the inherent chemome of a given HM should be eligible to act as the pronounced evidence for authentication. In particular, some so-called chemical markers, *e.g.*, artemisinin in *Artemisia annua* [10] and ginsenosides in *Panax plants* [11], primarily govern the plant chemotaxonomy position. More fortunately, a large portion of chemome can be successfully transferred from original plants to medicinal materials, and ultimately to the terminal products. For instances, ginsenosides frequently serve as the quality markers for those TCM prescriptions containing ginseng, notoginseng, or American ginseng [12]. In response to the decisive role of chemome towards authentication, diverse techniques, such as thin layer chromatography (TLC) imaging [13], LC fingerprinting [14] and LC-MS/MS-oriented chemical profiling [15], have been developed as workhorses for authentication through monitoring a set of compounds. Generally, when more compounds are involved in the diagnostic fingerprint chromatogram, greater confidence can be reached for herbal source authentication. Owing to the integration of the robust chromatographic potential from LC and the dramatical specific together with selective advantages from MS/MS, LC-MS/MS is now the most popular analytical tool to profile the chemome of HMs. Because of the extensive efforts made by the scientists worldwide, notably Chinese scientists, a huge size of LC-MS/MS data are available for HMs, notably those famous ones, such as *Ganoderma lucidum* [16], ginseng [17], Astragali Radix [18], and so forth, resulting in an annoying task for the application of those LC-MS/MS-dependent chemical profiles towards HMs authentication. In comparison of the high-level diversity for LC elution program, relatively lower variations occur, actually, for MS/MS measurements, and fortunately, MS/MS information instead of chromatographic behaviors plays the determinant role for chemical characterization. Therefore, when being applied alone, MS/MS dataset should be viable for HMs authentication if comparable, even identical, MS/MS information exists amongst different LC separations. LC is merely responsible for fractionating the entire HM extract and subsequently transferring to MS/MS equipment, and MS/MS information assignment and the subsequent structural identification task can be significantly alleviated. Due to the rapid development occurred for the scan rate and resolution properties of MS/MS in recent years, LC role should not affect MS/MS profile, theoretically, resulting in an opportunity to unify all existing LC-MS/MS information for a single HM.

Thereafter, HM authentication workload turns to pursue a superior route, rather than the conventional tables, to comprehensively exhibit MS/MS information. Binary code, barcode, as well as the emerging two-dimensional (2D) code are the three most popular ways for quick response, and especially, 2D-code is usually known as quick response tool and is advantageous at storing much more information than the other two available choices. In a previous study, we have proposed a versatile binary code strategy allowing sequence diagnostic compounds in most HMs [19]; however, the “coding-decoding” workflow is tightly dependent on a well-defined monitoring list, thus dampening the applicability. As an advanced study, here, we attempt to achieve the direct inter-conversion between MS/MS information and 2D-code, and moreover, those empirical mass fragmentation rules, the available databases, *e*.*g*., HMDB (https://hmdb.ca), Pubmed Compound (http://pubchem.ncbi.nlm.nih.gov), and ChemSpider (http://www.chemspider.com), as well as the relevant experiences can facilitate to translate 2D-code to chemical profile. The inter-habitat and inter-batch variations on the content patterns might impact the judgement of HM original sources and more fortunately, such influences can be omitted by 2D-code because the qualitative information of those primary components is merely taken into account. For applicability illustration and validation, in current study, *Ganodermalucidum* that is one of the most famous HMs underwent the “coding-decoding” workflow amongst MS/MS datafile, 2D-code, and chemical profile. We envision that the novel “2D-code” concept could exhibit the chemical profile in a quick response manner, and then aid rapid original source authentication and quality control of HMs.

## Two-dimensional code concept illustration

After LC–MS/MS measurements, the retention time (*t*R),MS$^1$, peak area, and MS$^2$ information is manually aligned to produce the dataset. The deduplication of those redundant MS$^1$ signals, such as [M+HCOO]$^{-}$, [M+Cl]$^{-}$, [M+Na]$^{+}$, [M+K]$^{+}$, and [M+NH$_4$]$^{+}$, is undertaken by paying special attention to those signals sharing completely identical *t*R values. Thereafter, all items are ranked by the peak area, and the *top-50* ones are merely taken into consideration. The *m/z* value of each deprotonated molecular signal is divided into integer and decimal portions. For a given *m/z* value, it exactly corresponds to the dot with *x* value being equal to integer portion and *y* value as 1000 times of decimal portion. In theory, all such dots are below the line of *y*=*x* because the decimal values are always lower than the integer portion after being multiplied by 1000. On the other side, *m/z* values of all fragment ions are exactly assigned as *x* values of the dots and the integer portions of the precursor ions are assigned as their *y* values. Regarding all fragment ion species of a given precursor ion, they are all distributed at line of *y* exactly being equal to *m/z* value of deprotonated molecular ion. Compared to that those dots corresponding to precursor ions are distributed at the bottom of *y*=*x* line, those fragment ion species frequently exist on the other side. As a result, 2D-code is thereafter formed by plotting these defined dots in the 2D-chart using Graphpad Prism 9.4.0 software (Graphpad, San Diego, CA).

The information corresponding to a deprotonated molecular ion is intrinsically scattered at the edges of each square frame. After extracting information, *m/z* values of both precursor ion and fragment ions can be translated to a compound through applying some empirical mass fragmentation rules and retrieving the available databases such as HMDB, Pubmed Compound, and ChemSpider. The entire code therefore corresponds exactly to a compound set.

**Fig. 1:**
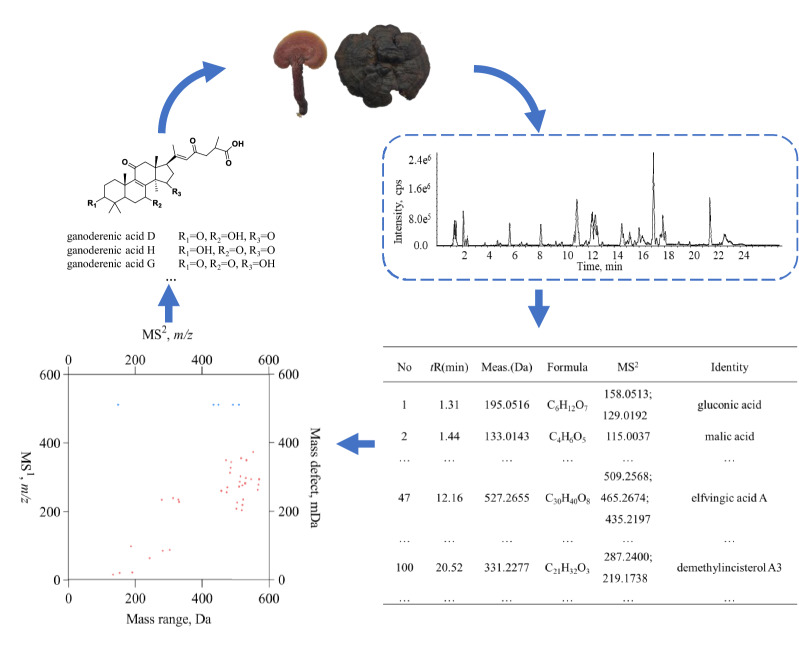
The “coding-decoding” flow-chart for 2D-code configuration of *Ganoderma* *lucidum*. The entire workflow was comprised of four consecutive steps: firstly, the extract of *Ganoderma* *lucidum* was analyzed with LC–MS/MS; secondly, the MS$^{1}$ and MS$^{2}$ spectra were carefully analyzed, aligned, and ranked using the peak area after redundant information deduplication; thirdly, MS$^{1}$ and MS$^{2}$ dataset for those *top-50* items was involved to plot the scattering dots by following the conversion rule described above, and eventually to produce the 2D-code; and fourthly, the chemical identification was made possible by the well-defined mass fragmentation and pertinent database

## Materials and methods

### Chemical and reagents

MS grade acetonitrile (ACN), methanol and formic acid were purchased from Thermo-Fisher (Pittsburgh, PA). Analytical grade methanol was obtained commercially from Beijing Chemical Works (Beijing, China). The ultra-pure water was prepared in-lab using a Milli-Q Integral water purification device (Milli-pore, MA). All materials related to *Ganoderma lucidum*, such as the fruit bodies (FGL) and the spore powders (SGL) of *Ganoderma lucidum* (Leyss. ex Fr.) Karst, and the fruit bodies of *Ganoderma sinense* J.D. Zhao, L.W. Hsu and X.Q. Zhang (FGS) were supplied by Xianzhilou Biotech Co. Ltd. (Nanping, China). A portion of FGL was further cut into stipes (sFGL) and pileus (pFGL) as well as FGS. The voucher specimens are deposited in the herbarium of our institute. Authentic compounds, including ganoderic acid A, were purchased from Yuanye Bio-Technology Co., Ltd. (Shanghai, China).

### Sample preparation

Each dried sample were individually pulverized. A 1.0 g aliquot of powders was sampled from each material batch and extracted with 50 mL methanol for 30 min in an ultrasonic-assisted manner. Afterwards, each extract was centrifuged at 12 000 rpm for 15 min and then the supernatant was filtered through a 0.22 $\mu$m Nylon membrane to generate the testing sample set.

### LC–MS/MS measurements

UHPLC (LC–20ADXR modular system, Shimadzu) coupled to a TripleTOF 6600${^+}$ mass spectrometer (SCIEX, Foster City, CA) was responsible for LC-MS/MS measurements. Chromatographic separations were performed on a reversed-phase HSS T3 column (2.1 mm $\times 100$ mm, 1.7 $\mu$m, Waters, USA). The mobile phase consisting of 0.1% aqueous of formic acid (A) and ACN (B) was programmed in gradient as follows: 0-20 min, 5%-95% B; 20-24 min, 95%-5% B and flow rate: 0.2 mL/min. The injection volume was set as 2 $\mu$L and the column oven was maintained at $40^{\circ }$C. Negative ionization polarity was applied. Collision energy (CE) was set as -35 eV and collision energy spread (CES) as 15 eV. The scan range for both MS$^1$ and MS$^2$ experiments ramped from *m*/*z* 100 to 1000 and the other parameters were set as follows: curtain gas, 35 psi; GS1, 50 psi; GS2, 50 psi; spray voltage, – 4500 V; and temperature, $500^{\circ }$C. SCIEX Analyst software (Version 1.6.2) was in charge of data processing and equipment synchronization.

### DI–MS/MS^ALL^ measurements

DI–MS/MS^ALL^ SCIEX TripleTOF 6600${^+}$ mass spectrometer (Foster City, CA) equipped with an ESI probe at 7 $\mu$L/min was used to analyze all samples including stipes and pileus of Ganoderma. All parameters were the same as those of in LC-Q-TOF-MS/MS measurements.

## Results and discussion

### Conversion from MS/MS dataset to 2D-code for *Ganoderma lucidum*

As aforementioned, the construction of 2D-code consisted of four consecutive steps (Fig. 1): first, the *Ganoderma lucidum* extract was measured with LC-MS/MS; second, MS$^1$ and MS$^2$ spectra were carefully processed, aligned, and ranked with the peak area after redundant information deduplication; third, *m/z* information of both deprotonated molecular ions and fragment ions for those *top-50* items was involved to plot the scattering dots by following the conversion rule described above, and ultimately to give birth to the 2D-code; and fourth, the well-defined mass fragmentation and relevant database retrieval enabled the chemical identification.

The fruit bodies of *Ganoderma lucidum* (FGL) for instance, FGL was firstly injected into LC–Q–TOF–MS/MS to acquire the mass spectral data in negative ionization mode. Thereafter, redundant information deduplication was conducted for MS$^1$ spectra to omit those adduct ions, *e*.*g*., [M+HCOO]$^{-}$, [M+Cl]$^{-}$, [M+Na]$^{+}$, [M+K]$^{+}$, and [M+NH$_4$]$^{+}$, and each rest precursor ion was assigned with MS$^2$ spectral signals and ranked with the peak area. Afterwards, the *top-50* items participated in 2D-code configuration. The integer portions and decimal portions multiplied by 1000 of *m/z* values for deprotonated molecular ions were assigned as *x* and *y* values for the dots, and on the other side, *m/z* value of each fragment ion was defined as *x* value and *m/z* values of the precursor ions served as *y* values for the dots belonging. 2D-code of FGL was ultimately generated by plotting all dots in a given 2D-chart. Then, the 2D-code was transmitted for structural annotation, in particular for the ganoderic acids because they serve as the primary effective chemical cluster for *Ganoderma lucidum*.

**Fig. 2:**
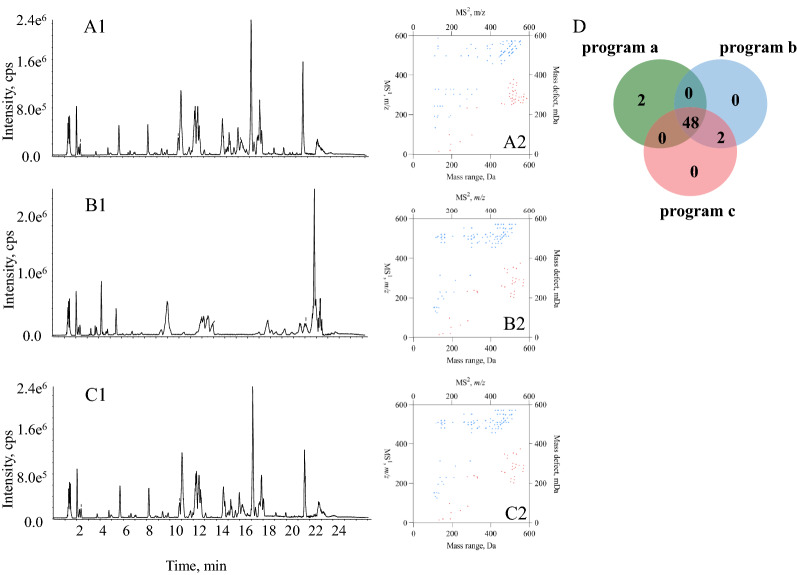
Comparison between different elution programs. Base Peak chromatograms of FGS in elution programs **a** (**A1**), **b** (**B1**) and **c** (**C1**). 2D-codes generated from three different mobile phase gradients (**A2**, **B2**, and **C2**) and Venn’s chart for the comparison amongst the chemical components involved in those 2D-code charts (**D**)

Regarding LC–MS/MS measurements, LC was intrinsically deployed to chromatographically fractionate the entire compound pool to sequential segments containing only several, even a single compound, prior to their entrances into MS/MS instrument that is responsible for analyte detection. In theory, the modifications of LC program cannot result in significant impact on the dataset consisting of items such as peak area, and MS$^1$ together with MS$^2$ spectral signals. In current study, evaluation was conducted for the impacts of chromatographic program modification towards the 2D-code outcomes. Three different elution programs (Programs a, b, and c with their base peak chromatograms in Fig. 2A1, B1 and C1, respectively) were applied for the chromatographic separations of the fruit bodies of *Ganoderma sinense* (FGS) that is a similar medicinal plant of *Ganoderma lucidum*, and the resultant MS/MS datasets were individually deployed to generate 2D-codes (Fig. 2A2, B2, and C2). Obviously, great similarity occurred amongst such codes from an identical sample. In-depth comparison was conducted by comparing the dots under *y*=*x* through employing Venn’s chart. As a result, full consistence occurred for the 2D-code between Programs b and c, and minor differences were observed between Programs a and b, or between Programs a and c, because 48 out of 50 components were shared by all 2D-codes and the other two components occurred as the unique feature for program a (Fig. 2D). Consequently, elution program could not significantly affect 2D-code, and such quick response tool was able to generically unify MS/MS dataset from different chromatographic program.

### Chemical characterization of chemical components by deciphering 2D-code

Through conducting the workflow, all primary MS$^1$ spectral information as well as all their fragment ions were implied in the 2D-code, and therefore, it should be quite convenient to translate the chart to a compound set through decoding those dots. As desired, totally identical 2D-code charts were obtained when conducting the inter- and intra-day assays, and moreover, the same charts were always formed for a selected sample although minor modifications occurred for the MS/MS settings. As aforementioned, all MS$^1$ spectral information was distributed under the line of *y*=*x*, whilst *m/z* values of those fragment ions could be found at the left region of the line. Exactly, the information belonging to a single compound, or an isomeric set, was distributed at the square frame constructed by lines of *x* being equal to the integer portion of precursor ion and *y* being equal to the exact *m/z* value of the deprotonated molecular ion. The empirical mass cracking rules together with accessible MS/MS databases subsequently facilitated to decode those values to chemical structures.

Taking the dot (*x*=459, *y*=275.2) that was obviously distributed under the line of *y* = *x* for instance, the molecular formula was calculated as C$_{27}$H$_{40}$O$_{6}$ (*m/z* 459.2752 [M-H]$^{-}$; error: 0.0 ppm). After paying attention to the region at the left of *y*=*x*, two dots bearing *x* values as 249.1512 and 209.1189 were distributed at the line of *y*=459.2752, indicating that signals at *m/z* 249.1512 and *m/z* 209.1189 should be the two fragment ions of *m/z* 459.2752 [M-H]$^{-}$. After retrieving these three key values in the compound set identified from *Ganoderma lucidum* [16, 20–24], lucidenic acid N came out as the sole candidate. Referring to the well-defined mass fragmentation pathways, the mass cracking channels being responsible for the generation of *m/z* 249.1512 and *m/z* 209.1189 are proposed in Additional file 1: Fig. S1.

**Fig. 3:**
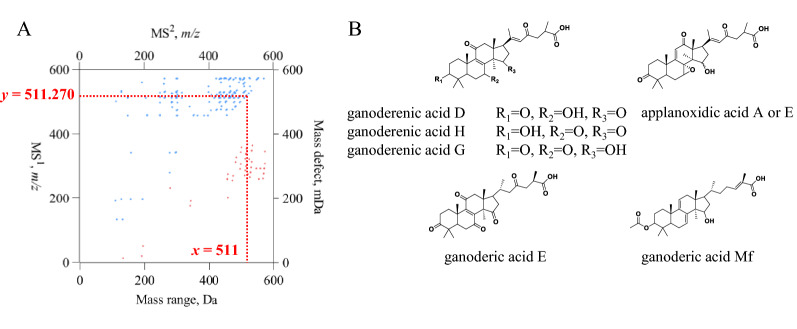
The utilization of “2D-code” concept towards chemical characterization of chemical components. 2D-code plot for *Ganoderma* *lucidum* and the dots captured by the square frame constructed by lines of *x*=511 and *y*=511.270 (**A**). Possible isomeric structures in respond to MS$^{1}$ spectral at *m*/*z* 511.270 and the dots distributed on the square frame (**B**)

Compared to that the dot (*x*=459, *y*=275.2) solely corresponds to a single structure candidate, sometimes, the occurrence of a given dot under the line of *y* = *x* might be jointly contributed by an isomeric set. Taking the [M-H]$^{-}$ signal (*x*=511, *y*=270.7) (Fig. 3A) for instance, the molecular formula was calculated as C$_{30}$H$_{40}$O$_{7}$ (*m/z* 511.2707 for [M-H]$^{-}$; error: 1.8 ppm). Ions at *m/z* 493.2631, 481.2254, 478.2449, 467.2831, 463.2132, 449.2728, 437.2357, 434.2479, 419.2253, 317.1763, 301.1801, 299.1658, 285.1881, 263.1301, 205.1234, and 149.0611 were found as the fragment ions for further structural identification. Based on databases and literatures, compounds that have been identified from Ganoderma genus bearing the chemical formula as C$_{30}$H$_{40}$O$_{7}$ include ganoderenic acid D, ganoderenic acid H, ganoderenic acid G, ganoderic acid E, ganoderic acid Mf, applanoxidic acid A and applanoxidic acid E (Fig. 3B).

The characteristic fragmentation pathways for these oxidative tetracyclic triterpenoids in negative ionization mode usually occur for the conjugated ring skeleton attributing to the different locations of hydroxyl groups and carbonyl groups at the tetracyclic skeleton. Guo, et al [16] summarized in depth the mass fragmentation behaviors and divided those tetracyclic triterpenoids into four groups as follows: 7-oxo-11-H or 7-oxo-11-hydroxy derivatives owning characteristic cleavage as ring A fission, 7-oxo-11-hydroxy derivatives showing characteristic cleavage as ring B fission, 7-hydroxy-15-oxo derivatives exhibiting characteristic cleavage of ring C fission, and oxidative tetracyclic triterpenoids of 11-oxo group possessing the cleavage as ring D fission due to the unstable five member ring. The above mass fragmentation patterns provided meaningful characteristic features for structural annotation even if the absence of chromatographic separation. Such mass fragmentation rules were subsequently applied to decipher the dots at the square frame.

Through analyzing the fragment ions of *m/z* 511, we found that the entire *m/z* value set could be divided into several groups. Ions at *m/z* 301 and 299 were assigned as the product ions resulted from the ring D fission which served as a dominant fragmentation route for ganoderic acid D, ganoderic acid E, ganoderenic acid G or ganoderenic acid H attributing to the occurrence of 11-oxo group. Ring D fission of ganoderenic acid D and ganoderenic acid H could yield fragment ion at *m/z* 301 whilst ganoderic acid E and ganoderenic acid G was able to produce the fragment ion at *m/z* 299. Since the C-ring fission was the characteristic dissociation pathway for 7-hydroxy-15-oxo derivatives, ganoderenic acid D was assigned to being responsible for the observation of ion at *m/z* 263 via ring C fission (Additional file 1: Fig. S2). In addition, fragment ions at *m/z* 478 and 463 were yielded from successive losses of methyl radicals at C-13 and C-14 sites, that is the featured fragmentation pathways for the compounds bearing 7,11-dioxo groups [16]. Therefore, fragment ions at *m/z* 478 and *m/z* 463 were tentatively assigned to ganoderic acid E, ganoderenic acid G or ganoderenic acid H. Accordingly, the sample should contain ganoderenic acid D, and moreover at least two ones in the group consisting of ganoderic acid E, ganoderenic acid G and ganoderenic acid H should also be involved in the sample. As expected, ganoderenic acid D, ganoderic acid E, ganoderenic acid G and ganoderic acid H were found when we employed the traditional workflow for chemical profile characterization (Additional file 1: Table S1), indicating that the 2D-code could generate comparable chemical profile outcome at the meanwhile of rapid data processing.

With this structural annotation strategy, a total of 49, 49, and 36 compounds were identified by similar decoding procedure in 2D-code of FGS, FGL and SGL, respectively. All the information is summarized in Additional file 1: Table S1. Because we primarily paid attention onto those primary MS$^1$ spectral signals, relatively less components were characterized in current study; however, in theory, the involvement of dozens of components is sufficient for authentication HMs because those abundant components usually own greater original authentication contributions in comparison of those less abundant, even trace compounds.

### 2D-code comparison amongst different materials

The present study primarily focused on the pursuit of a superior visible way to illustrate the chemome of HMs, and owing to the potential to store much information, 2D-code was employed *via* undertaking a smart data conversion approach. Because of the visible ability, chemome comparison should be convenient to achieve through directly mapping the 2D-codes. Thereafter, 2D-code comparison was carried out amongst different species and medicinal parts, such as the fruit bodies (FGL) and the spore powders (SGL) of *Ganoderma lucidum*, the fruit bodies of *Ganoderma* *sinense* (FGS), the stipes (sFGL) and pileus (pFGL) of FGL, the stipes (sFGS) and pileus (pFGS) of FGS. To achieve “one variable for one compound”, the dots under *y*=*x* were merely taken into consideration because deprotonated molecular ions of all primary components occurred on the right of *y*=*x*.

**Fig. 4:**
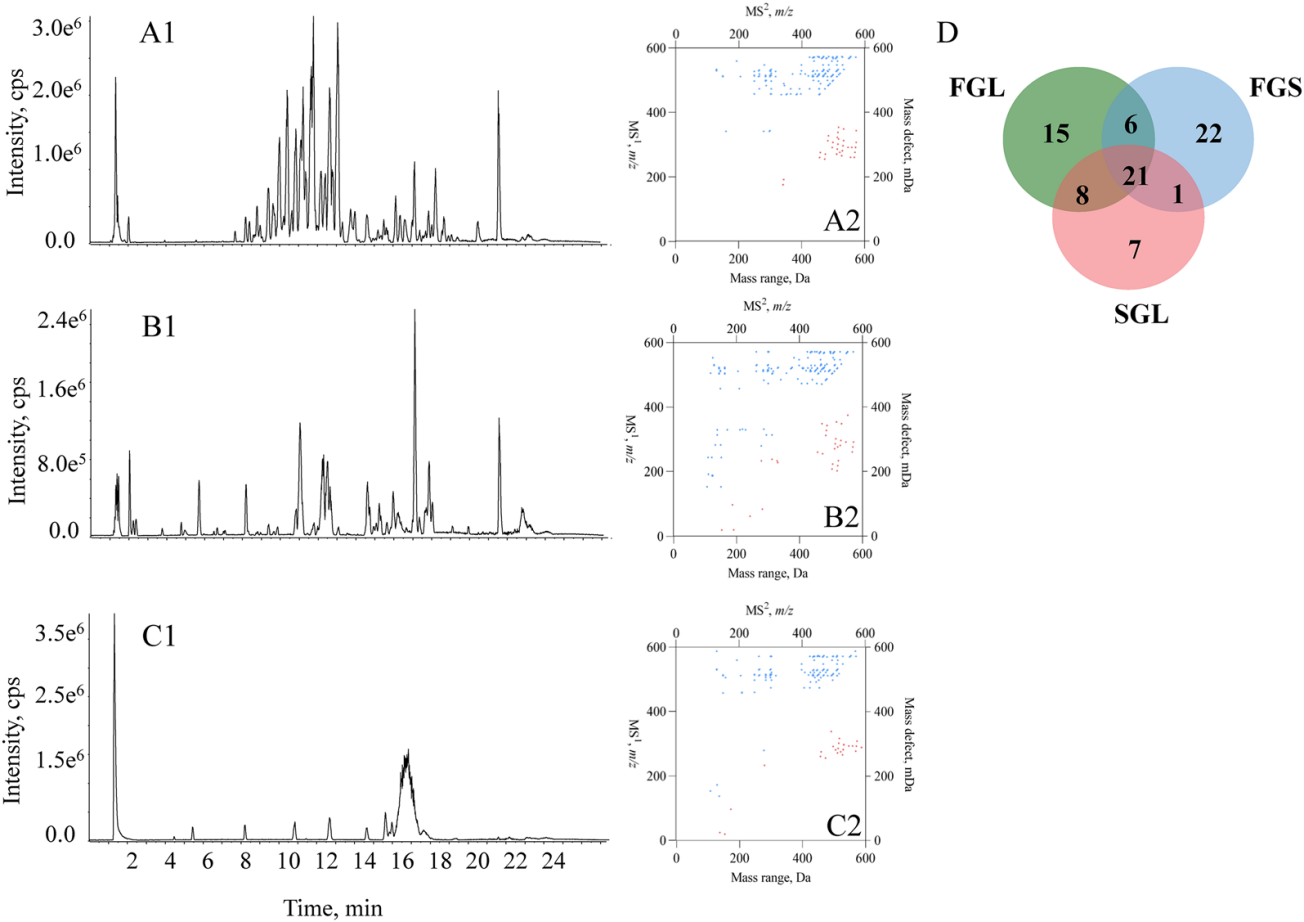
Comparison among FGL, FGS and SGL in LC–Q–TOF–MS/MS. Base Peak chromatograms of FGL (**A1**), FGS (**B1**) and SGL (**C1**); 2D-codes of FGL (**A2**), FGS (**B2**) and SGL (**C2**); Venn’s chart for the comparison amongst the chemical components involved in those 2D-code charts (**D**)

After applying the aforementioned workflow, MS/MS dataset of FGS, FGL and SGL were converted to 2D-code by involving the *top-50* MS$^1$ spectral signals (Fig. 4A1, B1, and C1). 2D-codes of FGS, FGL and SGL are illustrated as Fig. 4A2, B2, and C2. Noteworthily, owing to the removal of redundant information, 37 dots were only involved for SGL. Venn’s chart was employed to reach visible comparison that was undertaken for each pair, such as FGS *vs*. FGL, FGL *vs*. SGL, and FGS *vs*. SGL (Fig. 4D). Overall, even though FGS, FGL and SGL were usually treated as similar tonic materials, significant differences were observed amongst their 2D-codes. When referring to the compounds identified till now from Ganoderma genus, compounds bearing the molecular weight within the range of 100-300 Da are mainly flavonoids and organic acids, while most of the oxygenated tetracyclic triterpenoids have molecular weights amongst 400-600 Da [16, 25]. Regarding FGL, accumulation of dots belonging to the MS$^1$ spectral signals occurred in the range from *x* = 400 to *x* = 600, indicating that most of the primary chemical constituents in FGL were oxygenated tetracyclic triterpenoids (Figure 4A2). In the case of FGS, the dots corresponding to [M-H]$^{-}$ ions were sparsely distributed crossing the range from *x*=100 to *x*=300 (Fig. 4A2). Noteworthily, less components were observed in SGL compared to either FGL or FGS (Fig. 4C2), which was exactly consistent with the previous study [25]. Several studies have shown that the chemical compositions of FGS and FGL were significantly different, and the contents of ganoderic acids in FGS were usually lower than those in FGL [26–28]. After careful analysis of the LC-MS/MS dataset (Additional file 1: Table S1), we found that ganoderic acid B (*x*=515, *y*=303.2), ganoderic acid DM (*x*=513, *y*=323.4), ganoderic acid K (*x*=573, *y*=308.4), ganoderic acid F (*x*=545, *y*=275.6) were only present in the 2D-code of the FGL and nonetheless, absent in 2D-code of FGS. It was worthwhile to mention although ganoderic acid B was found in FGS by LC-MS/MS, none dot belonging to this compound was included in the 2D-code of FGS. Moreover, although being eligible to serve as the differential components between FGS and FGL, neither ganoderol B nor ganoderenic acid A (Additional file 1: Table S1) was observed in the 2D-code plots of FGS and FGL, attributing to their inferior distribution properties. On the contrary, ganoderic acid G (*x*=531, *y*=297.7), ganoderic acid A (*x*=515, *y*=301.8), ganoderic acid H (*x*=571, *y*=292.3) and ganoderenic acid D (*x*=511, *y*=271.7) were all present in the 2D-code plots of FGS and FGL, although they were sometimes regarded as less abundant components. Definitely, 2D-code provided a superior way to illustrate the chemical composition differences amongst the testing samples, and those differential compounds could be structurally identified through extracting *m/z* information from the coordinate values of those dots and translating them into structures.

To achieve visible comparison, Venn’s chart was applied to evaluate the differences amongst their 2D-code plots. Obviously, 21 components were shared by all FGS, FGL and SGL (Fig. 4D), and noteworthily, the component cluster accounted for 56.76% of the total 37 compounds in SGL. When comparing FGS and FGL that are derived from a single species, they shared 27 compounds, accounting for 54% of all compounds. Interestingly, a large portion of components (78.38% of the total 37 compounds) observed in SGL were also detectable in FGL; such 29 shared compounds merely accounted for 58% of the components involved in 2D-code of FGL. Regarding the comparison between FGS and SGL, the 22 shared compounds accounted for 44% and 59.46% of the compound profiles of FGS and SGL, respectively.

From the above comparison we could see that 2D-code contains so much MS data and can differentiate between different species of HMs just as well as other traditional methods of authentication. One of the common ways of identifying HMs is by appearance and taste. FGS has a purple-brown, purple-black to almost black pileus and a slightly bitter flavor. FGL has a reddish-brown, yellowish-brown to yellowish-brown pileus with pale white flesh and a very bitter flavor. However, if they became extracts or preparations, it was difficult to distinguish them from each other by appearance. When it came to the actual production and sale of Chinese medicine products, the production batch numbers currently in circulation are difficult to give consumers specific information about the preparations. It is not very feasible to use a list of ingredients on the label to represent HMs, either, as there are many ingredients that might take up large areas of paper. 2D-code enables cost effective tracking of sales logistics and promotion of products, and in our case, for traceability, authentication of HMs and determination of their main chemical components.

**Fig. 5:**
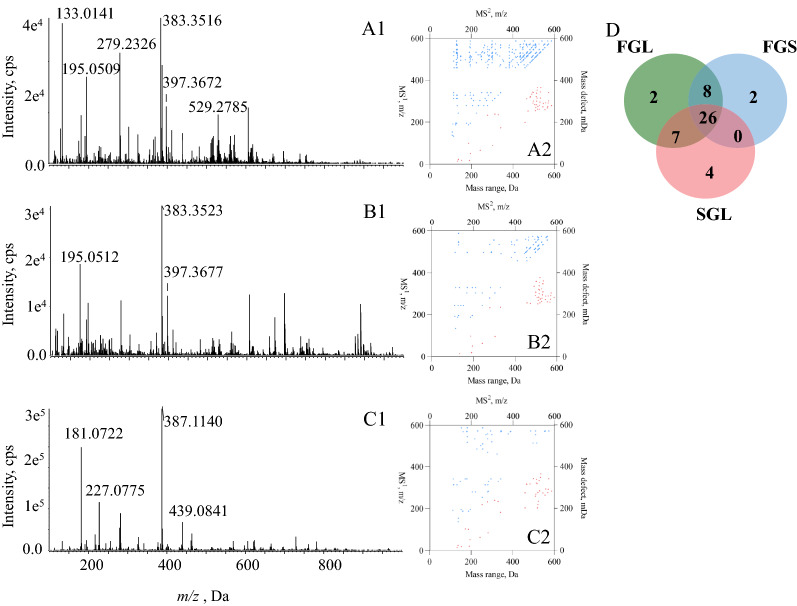
Comparison among FGL, FGS and SGL in DI–MS/MS^ALL^. MS^1^ spectrum of FGL (**A1**), FGS (**B1**) and SGL (**C1**); 2D-codes of FGL (**A2**), FGS (**B2**) and SGL (**C2**); Venn’s chart for the comparison amongst the chemical components involved in those 2D-code charts (**D**)

To build 2D-code in a more efficient manner, we also assayed the direct analysis of the extracts by DI–MS/MS$^\text{ALL}$, which was able to acquire all fragment ions by fragmenting the MS$^1$ spectral ion population into within consecutive narrow mass windows of 1 unit and had the advantage of high efficiency even being independent from liquid chromatographic separation. Because we have previously demonstrated elution program modifications could not significantly affect 2D-code, it should be theoretically feasible to apply DI-MS/MS$^\text{ALL}$ to produce 2D-codes aiming to rapid compare different species and medicinal parts of HMs. In current study, we injected FGL, FGS and SGL into DI-MS/MS$^\text{ALL}$ (Fig. 5A1, B1, and C1) and established their respective 2D-codes individually (Fig. 5A2, B2, and C2). As desired, totally identical 2D-code charts were obtained when conducting the inter- and intra-day assays for constructing 2D-codes by DI-MS/MS$^\text{ALL}$, and moreover, almost identical charts were formed for a selected sample although minor modifications occurred for the MS/MS settings. Moreover, similar 2D-code plots occurred for FGL, FGS or SGL when comparing the two analytical means such as DI-MS/MS$^\text{ALL}$ and LC-MS/MS, suggesting that 2D-code might be eligible to unify all chemical profile characterization results described previously, being regardless of the analytical methods. As expected, similar difference pattern observed by comparing 2D-codes yielded from LC-MS/MS also occurred when employing Venn’s chart to illustrate the difference properties in DI–MS/MS^ALL^. The ratio of the number of compounds in the inter-section to the overall number of components is as follows: FGS *vs*. SGL=55.32% , FGL *vs*. SGL = 70.21%, FGS *vs*. FGL = 75.56% (Fig. 5D). Overall, DI-MS/MS$^\text{ALL}$ yielded higher ratios compared to LC-MS/MS within different species resulting in more similar 2D-codes. Furthermore, between *x*=400 and *x*=600 below *y*=*x* we found concentrated clusters of dots in 2D-code constructed by DI-MS/MS$^\text{ALL}$. However, when the value of *m/z* was less than 400, 2D-code from DI-MS/MS$^\text{ALL}$ had more dots than that from LC-Q-TOF-MS/MS. The data generated using DI-MS/MS$^\text{ALL}$ were presumably selected from fragment ions within a 1 Da window of the first 50 abundances, implying that more data, especially more primary components were included, leading to the increment of similarity.

**Fig. 6:**
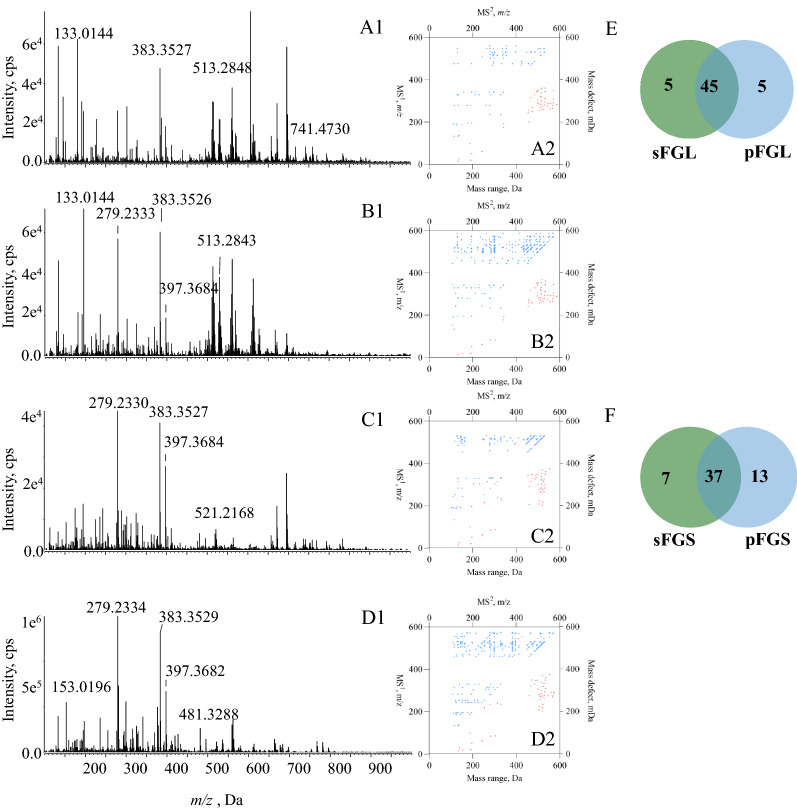
Comparison between sFGL and pFGL, sFGS and pFGS, respectively in DI–MS/MS^ALL^. MS$^{1}$ spectrum of sFGL (**A1**), pFGL (**B1**), sFGS (**C1**) and pFGS (**D1**); 2D-codes of sFGL (**A2**), pFGL (**B2**), sFGS (**C2**) and pFGS (**D2**). Venn’s chart for the comparison amongst the chemical components involved in 2D-code charts between sFGL and pFGL (**E**), sFGS and pFGS (**F**), respectively

Afterwards, we compared the chemical compositions of the different medicinal parts of FGL and FGS by constructing 2D-code by DI-MS/MS$^\text{ALL}$ (Fig. 6A1, B1, C1 and D1). Overall, there wasn’t any significant difference for different medicinal parts of FGL by directly visible comparison of 2D-code only (Fig. 6A2, B2 and E). Different dots in 2D-codes of sFGL and pFGL below *y*=*x* possessed coordinate values as *x*=137, *x*=187, *x*=455, *x*=475, *x*=153, *x*=282, *x*=303 and *x*=445. However, DI-MS/MS$^\text{ALL}$ didn’t detect any compound with the values of *m/z* greater than 500 below *y*=*x* in 2D-code of the sFGS (Fig. 6C2 and D2), indicating that FGS should be utilized with special attention in distinguishing between stipe and pileus. And there were significant differences in the 2D-codes of different medicinal parts of FGS although merely 37 compounds were involved (Fig. 6F). Different parts of Ganoderma were expected to own different pharmacological properties when being applied, owing to the differential chemical compositions [29]. 2D-code provided a possible solution for the identification of various parts of Ganoderma visually. In summary, 2D-code can be used to quickly obtain chemome profiles of different species and parts of *Ganoderma lucidum*, especially useful for distinguishing FGS from FGL or sFGS from pFGS in this case.

The “2D-code” concept exactly provided a meaningful visible approach to show the primary MS$^1$ and MS$^2$ spectral signals, thereafter, resulting in a chance to achieve rapid recognition through deciphering those dots to chemical structures. We regarded that the concept owned the advantages as follows:

i. 2D-code can visualize large amounts of MS/MS information in a rather small area.
ii. 2D-code can be used in a variety of forms including, but not limited to, raw herbal materials, prescriptions and extracts.
iii. 2D-code is suitable for use with a wide range of high-resolution mass spectrometers or related coupling devices.
iv. 2D-code simplifies the optimization of elution programs to reduce the injection time and should have good potential for high volume production.

On the other side, because the chromatographic properties were significantly suppressed when converting LC-MS/MS data to 2D-code, two primary drawbacks were generated as below:

i. Because 2D-codes doesn’t incorporate the values of retention time and the peak area, some isomers will inevitably be lost.
ii. The data conversion process is relatively cumbersome and a more convenient conversion process needs to be developed.

We have previously employed post-acquisition data processing techniques such as molecular weight imprinting (MWI) and mass defect filtering (MDF) to provide MS/MS data in a comparable two-dimensional chart. It was declaimed that the MDF properties of coumarins, saponins, xanthones, and oligosaccharide esters or their derivatives showed clustering trends [30, 31], indicating that a similar strategy to compare the broad dispersion of diverse chemome is conceivable. Actually, these robust post-acquisition data processing techniques are also applicable in 2D-code, because MS/MS spectral information was illustrated in the same way between 2D-code and DI-MS/MS$^\text{ALL}$. As traceability and accurate quality control at all stages of HMs manufacturing is becoming the future direction, a speedy, simple, and low-cost method is required to assure quality stability during production. Therefore, in current study we proposed a concept of 2D-code to exhibit the chemome of different species and parts of HMs based on the established workflow. Our study demonstrated that 2D-code had the advantages of compact size, broad applicability, and low time consumption and could be employed for source authentication and quality control of HMs.

## Conclusions

In current study, we proposed a novel means namely 2D-code to illustrate MS/MS dataset, and obviously, it was quite convenient to translate 2D-code to the chemical profile consisting of dozens of metabolites. After LC-MS/MS measurements of *Ganoderma* *lucidum*, the featured 2D-code was configured through fragmenting precursor ions to integer and decimal portions and assigning the integer parts as *y*-coordinate values of the fragment ions. A total of 50 primary compounds were tentatively characterized via deciphering the dots at the edges of each square frame. Through the comparison amongst different 2D-code plots, dramatical differences were found between different species and amongst different parts as well. More importantly, different chromatographic programs, even direct infusion, cannot significantly modify the appearance of 2D-code, suggesting that the concept is able to unify all chemical characterization outcomes for a single HM from different LC-MS/MS measurements. 2D-code should be a meaningful, practical visual way for rapid HM recognition because it is convenient to achieve the conversion amongst MS/MS dataset, 2D-code plot, and the chemome.

## Supplementary Information

**Additional file 1: Table S1.** Compounds identified from FGL, FGS and SGL in ESI negative mode using LC–Q-TOF-MS/MS. **Figure S1.** The proposed fragmentation pathways of lucidenic acid N. **Figure S2.** The proposed fragmentation pathways of ganoderenic acid D, ganoderic acid E, ganoderenic acid H and ganoderic acid G.

## Data Availability

The authors hereby declare that the data and materials in this study will be presented upon request from the corresponding author.
