# Supplementary material for: Two-dimensional code enables visibly mapping herbal medicine chemome: an application in Ganoderma lucidum
Source: Chin Med. 2023 Jan 12;18:6. doi: 10.1186/s13020-022-00702-8 (PMC9837956; doi:10.1186/s13020-022-00702-8)
Supplement: Supplementary file 1 — Additional file 1: Table S1. Compounds identified from FGL, FGS and SGL in ESI negative mode using LC–Q-TOF-MS/MS. Figure S1. The proposed fragmentation pathways of lucidenic acid N. Figure S2. The proposed fragmentation pathways of ganoderenic acid D, ganoderic acid E, ganoderenic acid H and ganoderic acid G. [file 13020_2022_702_MOESM1_ESM.docx]

Table S1 Compounds identified from FGL, FGS and SGL in ESI negative mode using LC–Q-TOF-MS/MS.

| No | *t*R  (min) | Pred.  (Da) | Meas.  (Da) | Error  (ppm) | Formula | MS^2^ | Identity | FGL | FGS | SGL |
| --- | --- | --- | --- | --- | --- | --- | --- | --- | --- | --- |
| 1 | 1.31 | 195.0510 | 195.0516 | 3.1 | C_6_H_12_O_7_ | 158.0513;129.0192 | gluconic acid | √ | – | – |
| 2 | 1.44 | 133.0142 | 133.0143 | 0.8 | C_4_H_6_O_5_ | 115.0037 | malic acid | √ | √ | – |
| 3 | 1.99 | 191.0197 | 191.0209 | 6.3 | C_6_H_8_O_7_ | 111.0086 | citric acid | √ | √ | – |
| 4 | 2.03 | 243.0623 | 243.0631 | 3.3 | C_9_H_12_N_2_O_6_ | 152.0353;122.0245;110.0245 | uridine isomer | – | √ | – |
| 5 | 2.36 | 282.0844 | 282.0849 | 1.8 | C_10_H_13_N_5_O_5_ | 150.0418;133.0155 | isoguanosine | – | √ | – |
| 6 | 4.47 | 153.0193 | 153.0195 | 1.3 | C_7_H_6_O_4_ | 109.0293 | 2,5-dihydroxybenzoic acid or isomer | – | – | √ |
| 7 | 5.43 | 137.0244 | 137.0247 | 2.2 | C_7_H_6_O_3_ | – | protocatechualdehyde | – | – | √ |
| 8 | 5.71 | 153.0193 | 153.0193 | 0.0 | C_7_H_6_O_4_ | 151.0036;107.0134 | 2,5-dihydroxybenzoic acid or isomer | – | √ | – |
| 9 | 6.49 | 303.0874 | 303.0871 | -1.0 | C_16_H_16_O_6_ | 159.0448;158.0360 | applanatin A isomer | – | √ | – |
| 10 | 6.79 | 172.0970 | 172.0974 | 2.3 | C_8_H_15_NO_3_ | 130.0880 | N-Acetyl-alloisoleucine | – | – | √ |
| 11 | 8.18 | 187.0976 | 187.0977 | 0.5 | C_9_H_16_O_4_ | 125.0966;123.0826 | azelaic acid | – | √ | – |
| 13 | 8.79 | 533.3120 | 533.3135 | 2.8 | C_30_H_46_O_8_ | 129.0555 | 12-hydroxyganoderic C2 or isomer | √ | – | – |
| 14 | 8.94 | 533.3120 | 533.3133 | 2.4 | C_30_H_46_O_8_ | 515.3048;303.1634;129.0556 | 12-hydroxyganoderic C2 or isomer | √ | – | – |
| 15 | 9.09 | 475.2701 | 475.2702 | 0.2 | C_27_H_40_O_7_ | 457.2594;439.2498 | lucidenic acid G or isomer | √ | √ | – |
| 16 | 9.39 | 527.2650 | 527.2668 | 3.4 | C_30_H_40_O_8_ | 509.2558;483.2776;465.2671;435.2182;317.1769;301.1837;249.1520 | 12-hydroxy-3,7,11,15,23-pentaoxo-lanost-8-en-26-oic acid | √ | – | – |
| 12 | 9.40 | 545.2756 | 545.2756 | 1.5 | C_30_H_42_O_9_ | 527.2674;509.2552; 483.2772; 483.2772;465.2661;317.1784;129.0557 | ganoderic acid F | √ | – | – |
| No | *t*R  (min) | Pred.  (Da) | Meas.  (Da) | Error  (ppm) | Formula | MS^2^ | Identity | FGL | FGS | SGL |
| 17 | 9.63 | 547.2913 | 547.2933 | 3.7 | C_30_H_44_O_9_ | 529.2850;511.2741;467.2828;399.2197 | 3*β*,7*β*,12-trihydroxy-4(hydroxymethyl)-11,15,23trioxolanost-8-en-26-oic acid | √ | √ | √ |
| 18 | 10.00 | 531.2963 | 531.2965 | 0.4 | C_30_H_44_O_8_ | 129.0561 | ganoderic acid I | √ | √ | √ |
| 19 | 10.22 | 457.2596 | 457.2593 | -0.7 | C_27_H_38_O_6_ | 365.1638;303.1978;249.1505 | 3-hydroxy-4,4,14-trimethyl-7,11,15-trioxochol-8-en-24-oic-acid | √ | √ | – |
| 20 | 10.30 | 571.2913 | 571.2925 | 2.1 | C_32_H_44_O_9_ | 529.2819;511.2710;499.2349;497.2514;467.2844;455.2455;455.2447;440.2218;437.2344;425.1984 | butyl lucidenate E2 | √ | √ | √ |
| 21 | 10.35 | 517.3171 | 517.3167 | -0.8 | C_30_H_46_O_7_ | 499.3080 | lucidenic acid P or isomer | √ | – | √ |
| 22 | 10.60 | 527.2650 | 527.2647 | -0.6 | C_30_H_40_O_8_ | 509.2582;479.2450;447.2536 | 3,12-dihydroxy-4,4,14-trimethyl-7,11,15-trioxo-lanost-8,9,20,22-en-26-oic acid | √ | – | √ |
| 23 | 10.60 | 521.2756 | 521.2753* | -0.6 | C_27_H_40_O_7_ | 475.2723;457.2627;439.2498;427.2142;409.2050;303.1974 | lucidenic acid G or isomer | √ | √ | √ |
| 24 | 10.62 | 531.2963 | 531.2974 | 2.1 | C_30_H_44_O_8_ | 513.2903;469.2984;301.1835 | iso-ganoderic acid G | √ | √ | – |
| 25 | 10.64 | 475.2701 | 475.2711 | 2.1 | C_27_H_40_O_7_ | 439.2532;427.2130;303.1997 | lucidenic acid G or isomer | √ | – | – |
| 26 | 10.65 | 587.2844 | 587.2885 | 7.0 | C_32_H_44_O_10_ | 569.2797;129.0565 | 3*β*,7*β*-dihydroxy-12*β*-acetoxy-11,15,23-trioxo-5α-lanosta-8-en-26-oic acid methyl ester or isomer | – | – | √ |
|  |  |  |  |  |  |  |  |  |  |  |
| No | *t*R  (min) | Pred.  (Da) | Meas.  (Da) | Error  (ppm) | Formula | MS^2^ | Identity | FGL | FGS | SGL |
| 28 | 10.80 | 529.2807 | 529.2822 | 2.8 | C_30_H_42_O_8_ | 511.2742;481.2269;467.2834;437.2356 | ganoderic acid C6 or isomer | √ | √ | √ |
| 29 | 10.80 | 459.2752 | 459.2752 | 0.0 | C_27_H_40_O_6_ | 249.1512;209.1189 | lucidenic acid N | √ | – | √ |
| 30 | 10.81 | 511.2701 | 511.2717 | 3.1 | C_30_H_40_O_7_ | 511.2734;481.2254;467.2831;437.2357 | ganoderenic acid D | √ | √ | √ |
| 31 | 10.93 | 457.2596 | 457.2597 | 0.2 | C_27_H_38_O_6_ | 457.2610;397.2360;385.2374;353.2494;301.1845;249.1507 | dehydrolucidenic acid N | √ | – | – |
| 32 | 11.04 | 513.2858 | 513.2870 | 2.3 | C_30_H_42_O_7_ | 495.2777;469.2978;451.2682;301.1815;265.1452;249.1503 | ganoderenic acid A | √ | – | √ |
| 33 | 11.05 | 329.2334 | 329.2344 | 3.0 | C_18_H_34_O_5_ | 229.1457;211.1348;171.1028;139.1125 | tricin | – | √ | – |
| 34 | 11.08 | 531.2963 | 531.2977 | 2.6 | C_30_H_44_O_8_ | 513.2884;469.2978;301.1822;265.1452 | ganoderic acid G | √ | √ | √ |
| 36 | 11.21 | 515.3014 | 515.3002 | -2.3 | C_30_H_44_O_7_ | 497.2945;453.3027;303.1985;249.1513 | ganoderic acid B | √ | √ | √ |
| 37 | 11.38 | 529.2807 | 529.2817 | 1.9 | C_30_H_42_O_8_ | 511.2728;399.2206;129.0562 | unknown | √ | √ | √ |
| 38 | 11.41 | 571.2913 | 571.2923 | 1.8 | C_32_H_44_O_9_ | 553.2830;511.2677;467.2815;449.2734;437.2309;303.1966;301.1806 | ganoderic acid H | √ | √ | √ |
| 39 | 11.45 | 559.2913 | 559.2931* | 3.2 | C_30_H_42_O_7_ | 513.2888;495.2762;439.2506;193.0878 | ganoderenic acid A isomer | √ | – | √ |
| 40 | 11.45 | 573.3069 | 573.3084 | 2.6 | C_32_H_46_O_9_ | 555.2996;511.3107;469.3000;451.2869 | ganoderic acid K | √ | – | √ |
| 41 | 11.59 | 505.2807 | 505.2810* | 0.6 | C_27_H_40_O_6_ | 459.2718;441.2636;300.1740;285.1493;157.0901 | lucidenic acid Q | – | – | √ |
| 42 | 11.63 | 571.2913 | 571.2935 | 3.9 | C_32_H_44_O_9_ | 553.2846;511.2740;467.2831;437.2348 | ganoderenic acid K or isomer | √ | √ | √ |
| 43 | 11.75 | 515.3014 | 515.3018 | 0.8 | C_30_H_44_O_7_ | 497.2937;453.3042;435.2911;300.1718;301.1822;299.1620;285.1522 | ganoderic acid A | √ | √ | √ |
| No | *t*R  (min) | Pred.  (Da) | Meas.  (Da) | Error  (ppm) | Formula | MS^2^ | Identity | FGL | FGS | SGL |
| 44 | 11.86 | 529.2807 | 529.2802 | -0.9 | C_30_H_42_O_8_ | 511.2740;467.2815;449.2718;299.1656;263.1295 | ganoderic acid M | √ | √ | √ |
| 45 | 11.97 | 473.2545 | 473.2556 | 2.3 | C_27_H_38_O_7_ | 455.2464;425.2975;407.1879;301.1814 | lucidenic acid B | √ | √ | √ |
| 46 | 11.99 | 553.3746 | 553.3726* | -3.6 | C_30_H_52_O_6_ | 507.3718;427.1780;383.1870;125.0235 | unknown | – | √ | – |
| 47 | 12.16 | 527.2650 | 527.2655 | 0.9 | C_30_H_40_O_8_ | 509.2568;465.2674;435.2197 | elfvingic acid A | √ | – | √ |
| 48 | 12.20 | 523.2337 | 523.2342 | 1.0 | C_30_H_36_O_8_ | 479.2464;377.1966;333.2083;163.0406;145.0300 | unknown | – | √ | – |
| 49 | 12.23 | 521.2181 | 521.2187 | 1.2 | C_30_H_34_O_8_ | 477.2295;375.1810;331.1903;163.0403;145.0294 | unknown | – | √ | – |
| 50 | 12.24 | 457.2596 | 457.2606 | 2.2 | C_27_H_38_O_6_ | 209.1194;149.0608 | lucidenic acid A | √ | √ | √ |
| 51 | 12.38 | 529.2807 | 529.2815 | 1.5 | C_30_H_42_O_8_ | 511.2733;263.1307 | 12-hydroxyganoderic acid D | √ | √ | √ |
| 52 | 12.41 | 519.2024 | 519.2034 | 1.9 | C_30_H_32_O_8_ | 475.2154;457.2121;411.1833;373.1649;311.1655;163.0407;145.0301 | unknown | – | √ | – |
| 53 | 12.42 | 511.2701 | 511.2710 | 1.8 | C_30_H_40_O_7_ | 493.2631;467.2822;449.2728;434.2498;317.1763;299.1658;285.1881;263.1301;205.1234;149.0611; | ganoderic acid E | √ | √ | √ |
| 54 | 12.57 | 513.2858 | 513.2859 | 0.2 | C_30_H_42_O_7_ | 495.2767;451.2858;301.1812;149.0611 | ganoderic acid AM1 | √ | √ | √ |
| 55 | 12.64 | 521.2181 | 521.2191 | 1.9 | C_30_H_34_O_8_ | 477.2308;413.1970;375.1809;331.1915;163.0406;145.0299 | unknown | – | √ | – |
| 56 | 12.74 | 511.2701 | 511.2717 | 3.1 | C_30_H_40_O_7_ | 493.2629;449.2726;434.2479;299.1658;285.1509;149.0608 | ganoderenic acid G | √ | √ | √ |
| No | *t*R  (min) | Pred.  (Da) | Meas.  (Da) | Error  (ppm) | Formula | MS^2^ | Identity | FGL | FGS | SGL |
| 57 | 12.84 | 571.2913 | 571.2938 | 4.4 | C_32_H_44_O_9_ | 553.2853;511.2723;467.2843;449.2733;300.1753;263.1305 | ganodernoid G or isomer | √ | √ | √ |
| 58 | 12.87 | 499.3065 | 499.3071 | 1.2 | C_30_H_44_O_6_ | 481.2951;437.3080;287.2035;285.1873 | ganolucidic acid A | √ | – | – |
| 59 | 12.90 | 567.2600 | 567.2620 | 3.5 | C_32_H_40_O_9_ | 549.2548;507.2399;499.3092;477.1944 | ganodernoid D | √ | √ | – |
| 60 | 13.04 | 569.2756 | 569.2770 | 2.5 | C_32_H_42_O_9_ | 551.2684;509.2573;479.2085;465.2667;435.2181 | 12-acetoxyganoderic acid | √ | √ | √ |
| 61 | 13.32 | 513.2858 | 513.2868 | 1.9 | C_30_H_42_O_7_ | 495.2796;451.2868;421.2406;301.1836;285.1524 | ganoderic acid J | √ | √ | √ |
| 62 | 13.32 | 533.3484 | 533.3485* | 0.2 | C_30_H_48_O_5_ | 487.3392 | leucocontextin W or isomer | √ | √ | – |
| 63 | 13.37 | 567.2600 | 567.2621 | 3.7 | C_32_H_40_O_9_ | 549.2537;507.2412;477.1934;463.2498 | iso-ganodernoid D | √ | √ | – |
| 64 | 13.43 | 511.2701 | 511.2712 | 2.2 | C_30_H_40_O_7_ | 493.2588;478.2449;463.2132;449.2726;437.2385;434.2477;419.2253;301.1801;299.1652; | ganoderenic acid H | √ | – | – |
| 65 | 13.71 | 555.2600 | 555.2602* | 0.4 | C_30_H_38_O_7_ | 509.2560;261.1143 | ganoderenic acid F | √ | – | – |
| 66 | 13.71 | 533.3484 | 533.3484* | 0.0 | C_30_H_48_O_5_ | 487.3446;469.3338 | leucocontextin W or isomer | √ | √ | – |
| 67 | 13.99 | 535.3640 | 535.3649* | 1.7 | C_30_H_50_O_5_ | 489.3555 | ganoderiol H | √ | – | – |
| 68 | 14.38 | 499.3065 | 499.3059 | -1.2 | C_30_H_44_O_6_ | 455.3255;401.2358;285.1871 | ganolucidic acid D | √ | – | – |
| 69 | 14.46 | 533.3484 | 533.3490* | 1.1 | C_30_H_48_O_5_ | 487.3329 | leucocontextin W or isomer | √ | √ | – |
| 70 | 14.53 | 497.2909 | 497.2909 | 0.0 | C_30_H_42_O_6_ | 479.2815;435.2949 | ganoderic acid GS-1 isomer | √ | – | – |
|  |  |  |  |  |  |  |  |  |  |  |
| No | *t*R  (min) | Pred.  (Da) | Meas.  (Da) | Error  (ppm) | Formula | MS^2^ | Identity | FGL | FGS | SGL |
| 71 | 14.72 | 505.2232 | 505.2256 | 4.8 | C_30_H_34_O_7_ | 461.2366;359.1894;315.1980;163.0402;145.0301;118.0424 | unknown | – | √ | – |
| 72 | 14.74 | 459.2024 | 459.2024 | 0.0 | C_24_H_30_O_6_ | 354.9922 | 8*α*,9*α*-epoxy-4,4, 14*α*-trimethyl-3,7,11,15,20-pentaoxo-5α-pregnane | √ | – | – |
| 73 | 15.08 | 313.2384 | 313.2389 | 1.6 | C_18_H_34_O_4_ | 295.2286;139.1129 | (+/-)12,13-Dihome | – | √ | – |
| 74 | 15.10 | 533.3484 | 533.3484* | 0.0 | C_30_H_48_O_5_ | 487.3364 | leucocontextin W or isomer | √ | – | – |
| 75 | 15.19 | 483.3116 | 483.3127 | 2.3 | C_30_H_44_O_5_ | 287.2039 | dehydrolucidenic acid N isomer | √ | √ | – |
| 76 | 15.23 | 503.2075 | 503.2079 | 0.8 | C_30_H_32_O_7_ | 459.2191;313.1805;163.0396;145.0297 | unknown | – | √ | – |
| 77 | 15.43 | 343.1915 | 343.1917 | 0.6 | C_21_H_28_O_4_ | 299.2033 | ganomycin B | √ | – | – |
| 78 | 15.74 | 515.3378 | 515.3390* | 2.3 | C_30_H_46_O_4_ | 469.3334 | ganoderiol B or isomer | √ | – | – |
| 79 | 16.14 | 341.1758 | 341.1760 | 0.6 | C_21_H_26_O_4_ | 297.1869;279.1778;159.0447 | ganofuran B | √ | – | – |
| 80 | 16.20 | 487.3429 | 487.3439 | 2.1 | C_30_H_48_O_5_ | 395.3349 | leucocontextin W or isomer | – | √ | – |
| 81 | 16.39 | 519.3691 | 519.3698* | 1.3 | C_30_H_50_O_4_ | – | ganoderiol A | √ | – | – |
| 82 | 16.46 | 517.3535 | 517.3535* | 0.0 | C_30_H_48_O_4_ | 471.3484 | unknown | – | √ | – |
| 83 | 16.64 | 487.3429 | 487.3429 | 0.0 | C_30_H_48_O_5_ | 487.3455 | leucocontextin W or isomer | – | √ | – |
| 84 | 16.99 | 485.3273 | 485.3273 | 0.0 | C_30_H_46_O_5_ | 485.3289 | 3*β*,15*α*,22*β*-trihydroxylanosta-7,9(11),24-trien-26-oic acid | √ | √ | – |
| 83 | 16.64 | 487.3429 | 487.3429 | 0.0 | C_30_H_48_O_5_ | – | leucocontextin W or isomer | – | √ | – |
| 84 | 16.99 | 485.3273 | 485.3273 | 0.0 | C_30_H_46_O_5_ | – | 3*β*,15*α*,22*β*-trihydroxylanosta-7,9(11),24-trien-26-oic acid | √ | √ | – |
| No | *t*R  (min) | Pred.  (Da) | Meas.  (Da) | Error  (ppm) | Formula | MS^2^ | Identity | FGL | FGS | SGL |
| 85 | 17.24 | 525.3222 | 525.3220 | -0.4 | C_32_H_46_O_6_ | 483.3155;287.2029;285.1872 | 3,7-oxo-12-acetylganoderic acid DM or isomer | √ | – | – |
| 86 | 17.29 | 517.3535 | 517.3535* | 0.0 | C_30_H_48_O_4_ | 471.3430 | unknown | √ | – | – |
| 87 | 17.33 | 485.3273 | 485.3277 | 0.8 | C_30_H_46_O_5_ | 441.3388;341.2517 | 3*α*,16*α*,26-trihydroxylanosta-7,9 (11),24-trien-21-oic acid | √ | √ | – |
| 88 | 17.38 | 515.3378 | 515.3386* | 1.6 | C_30_H_46_O_4_ | 469.3359 | ganoderiol B or isomer | √ | – | – |
| 89 | 17.57 | 517.3535 | 517.3547* | 2.3 | C_30_H_48_O_4_ | 471.3543 | unknown | √ | √ | – |
| 90 | 17.69 | 573.3433 | 573.3456* | 4.0 | C_32_H_48_O_6_ | 527.3417;485.3303 | ganoderic acid V | √ | – | – |
| 91 | 18.20 | 517.3535 | 517.3550* | 2.9 | C_30_H_48_O_4_ | 471.3465 | unknown | √ | √ | – |
| 92 | 18.53 | 515.3378 | 515.3379* | 0.2 | C_30_H_46_O_4_ | 469.3343 | ganoderiol B or isomer | √ | – | – |
| 93 | 18.53 | 501.3586 | 501.3585* | -0.2 | C_30_H_48_O_3_ | 455.3581 | ganodermatriol or isomer | √ | – | – |
| 94 | 18.68 | 513.3222 | 513.3234* | 2.3 | C_30_H_44_O_4_ | 467.3196 | ganoderic acid DM | √ | – | – |
| 95 | 19.11 | 491.3367 | 491.3381* | 2.9 | C_28_H_46_O_4_ | 445.3362;427.3208 | petchinoids B | – | – | – |
| 96 | 19.82 | 501.3586 | 501.3587* | 0.2 | C_30_H_48_O_3_ | 112.9841 | ganodermatriol or isomer | √ | – | – |
| 97 | 19.94 | 471.3480 | 471.3486 | 1.3 | C_30_H_48_O_4_ | – | lucidumol A | – | √ | – |
| 98 | 20.16 | 499.3429 | 499.3436* | 1.4 | C_30_H_46_O_3_ | 453.3331 | ganoderal B | √ | – | – |
| 99 | 20.16 | 541.3535 | 541.3507* | -5.2 | C_32_H_48_O_4_ | 495.3506;467.3466;440.3335;380.3183 | 3*β*-hydroxy-15*α*-acetoxy-5*α*-lanosta-7,9(11),24-trien-26-al | – | – | – |
| 100 | 20.52 | 331.2279 | 331.2277 | -0.6 | C_21_H_32_O_3_ | 287.2400;219.1738 | demethylincisterol A3 | – | √ | – |
| 101 | 21.64 | 279.2330 | 279.2335 | 1.8 | C_18_H_32_O_2_ | – | linoleic acid | √ | √ | – |


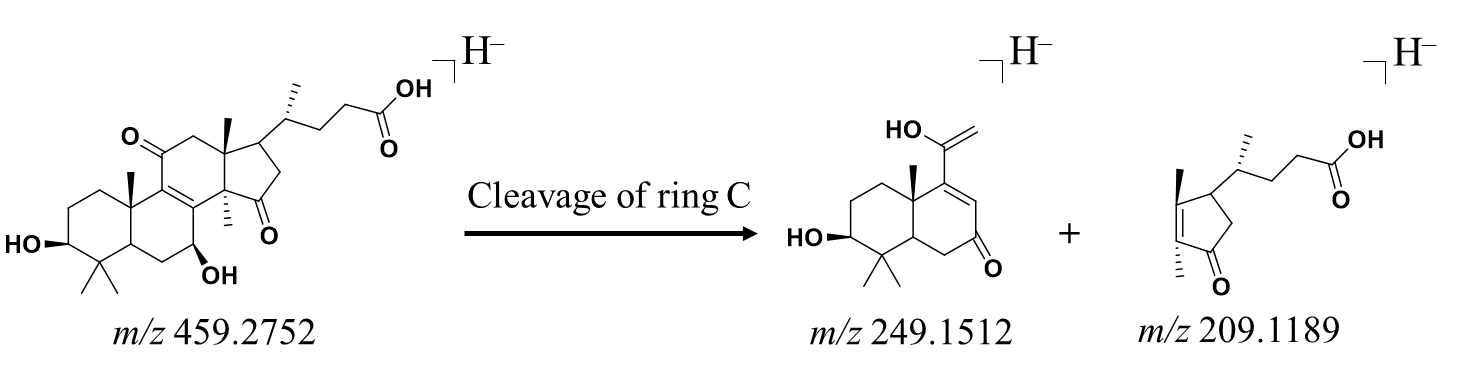


Figure S1 The proposed fragmentation pathways of lucidenic acid N


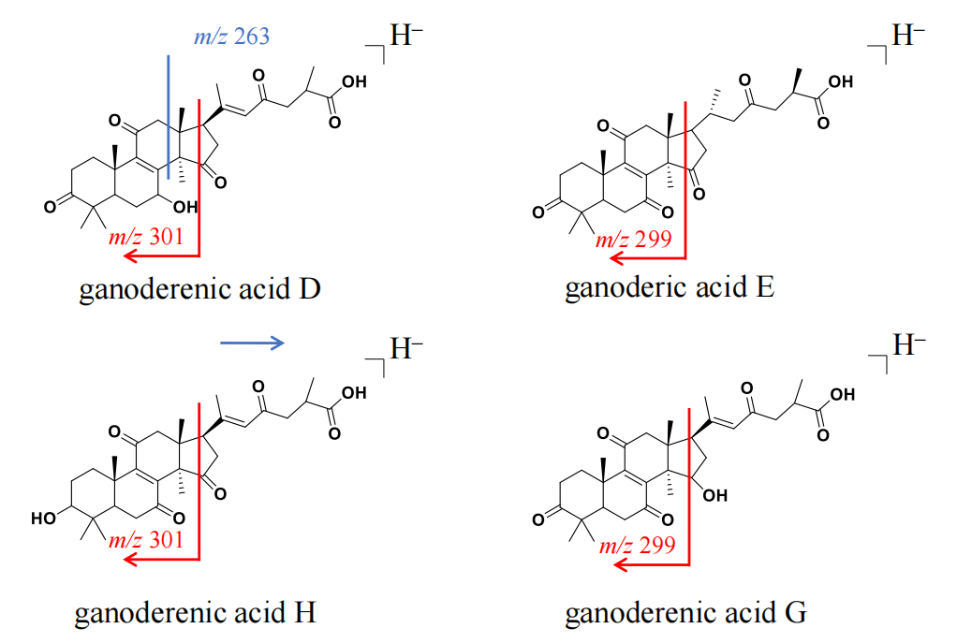


Figure S2 The proposed fragmentation pathways of ganoderenic acid D, ganoderic acid E,

ganoderenic acid H and ganoderic acid G.
